# Olive Mill Waste Enhances α-Glucan Content in the Edible Mushroom *Pleurotus eryngii*

**DOI:** 10.3390/ijms18071564

**Published:** 2017-07-18

**Authors:** Sharon Avni, Nirit Ezove, Hilla Hanani, Itamar Yadid, Michal Karpovsky, Hilla Hayby, Ofer Gover, Yitzhak Hadar, Betty Schwartz, Ofer Danay

**Affiliations:** 1 Edible Mushrooms Development, MIGAL, Kiryat Shmona 11016, Israel; sharony20@gmail.com (S.A.); niritezov@walla.co.il (N.E.); lilohila90@gmail.com (H.H.); itamarya@migal.org.il (I.Y.); 2 Tel Hai College, Upper Galilee 12210, Israel; 3 Institute of Biochemistry, School of Nutritional Sciences, Food Science and Nutrition, The Robert H. Smith Faculty of Agriculture, Food and Environment, The Hebrew University of Jerusalem, Rehovot 76100, Israel; michal.karpob@gmail.com (M.K.); hilla.hayby@mail.huji.ac.il (H.H.); ofer.gover@mail.huji.ac.il (O.G.); 4 Department of Plant Pathology and Microbiology, The Robert H. Smith Faculty of Agriculture, Food and Environment, The Hebrew University of Jerusalem, Rehovot 76100, Israel; yitzhak.hadar@mail.huji.ac.il

**Keywords:** α-glucans, β-glucans, *Pleurotus* species, *Pleurotus eryngii*, olive mill solid waste (OMSW)

## Abstract

Mushroom polysaccharides are edible polymers that have numerous reported biological functions; the most common effects are attributed to β-glucans. In recent years, it became apparent that the less abundant α-glucans also possess potent effects in various health conditions. Here we explore several *Pleurotus* species for their total, β and α-glucan content. *Pleurotus eryngii* was found to have the highest total glucan concentrations and the highest α-glucans proportion. We also found that the stalks (stipe) of the fruit body contained higher glucan content then the caps (pileus). Since mushrooms respond markedly to changes in environmental and growth conditions, we developed cultivation methods aiming to increase the levels of α and β-glucans. Using olive mill solid waste (OMSW) from three-phase olive mills in the cultivation substrate. We were able to enrich the levels mainly of α-glucans. Maximal total glucan concentrations were enhanced up to twice when the growth substrate contained 80% of OMSW compared to no OMSW. Taking together this study demonstrate that *Pleurotus eryngii* can serve as a potential rich source of glucans for nutritional and medicinal applications and that glucan content in mushroom fruiting bodies can be further enriched by applying OMSW into the cultivation substrate.

## 1. Introduction

Mushrooms have a long history of consumption as food, and also have remarkable therapeutic properties which are mainly recognized in oriental countries [1,2]. Many of the basidiomycetes produce polysaccharides with very important advantageous medicinal properties, such as anti-cancer and immunomodulation properties [3,4], thus enhancing their content could improve the value of the mushrooms.

Glucans (C_6_H_12_O_5_)_n_ are polysaccharides built of glucose monosaccharides linked by α, β, or mixed α-β glycosidic bonds. β-d-glucans are a naturally-occurring structural component of the cell walls of the fungal mycelium [4,5]. Nowadays, *Pleurotus* genus’s (Oyster) pleuran is one of the most important and effective sources of β-glucans [6]. This compound is built of molecules of glucose linked by (1→3)-β bonds. This backbone with a linear structure is linked with side-chains built of glucopyranose molecules. Every four molecules of glucose in the backbone are connected with 0 to 6 molecules of glucopyranose as side-chains [7].

In contrast to (1→3)-β-glucan, there are limited reports on the structure of α-glucans, possibly due to their low concentration in mushrooms and uncertain biological function. However, a few studies report that alkaline-soluble α-(1→3)-d-glucan is the main α-glucan structure in shiitake and Oyster mushrooms [8,9,10].

Mushrooms contain consistently high levels of β-glucans, on average 30–40% by weight (*w*/*w*). However, α-glucans content is much lower. Among four different *P. ostreatus* strains, the α-glucan content was 3.4–7.9% in the pileus and 3.0–7.6% in stems, compared to 27.4–39.2% β-glucans in the pileus and 35.5–50.0% in the stems [11]. It is worth mentioning that only few studies have quantified α-glucans, compared to many studies that examined and quantified the β-glucan content of mushrooms. Thus, further studies that examine α-glucan content of mushrooms will expand knowledge regarding the α-glucan content and their putative health benefit activities.

β-glucans are non-digestible carbohydrates with potent immunomodulatory effects on both innate and adaptive immunity [6,12]. However, β-glucans in mushrooms differ in structure, water solubility, molecule size, and molecular mass, thus not all fungal β-glucans show equally strong healing activity [13,14,15]. The effect of β-glucan activity is also determined by their solubility [16], isolation method, and origin, which influence the diversity of their structure and degree of polymerization and thereby their molecular mass [7].

Compared with the intensive investigations of β-d-glucans, the biological activity of α-(1→3)-d-glucan has been reported only rarely in the literature. Recently, more attention has been given to sulfation, sulfonylation, and carboxymethylation of α-glucan polysaccharides which increase their solubility and antitumor activity [10,17]. Additionally, our previous studies emphasize that α glucans are molecules exerting prominent anticancer and anti-inflammatory activities and this glucan fraction and are amenable to extraction in a water-soluble phase [18,19,20].

There has been growing popularity in developing mushroom glucans for medicinal applications or as dietary supplements [21]. Mushrooms react with extreme sensitivity to changes in their environmental conditions. Therefore, we assumed that it is possible to increase α and β glucan content by changing the substrate composition. In this regard, a previous study [22] demonstrated that growing the edible mushroom *Pleurotus ostreatus* in a substrate containing 10% of dry olive mill solid waste (OMSW) improved the nutritive value of the treated OMSW for ruminant farm animals. It was shown that while growing on OMSW *P. ostreatus* utilized the lipid-soluble ingredients of the OMSW. The enhanced utilization of lipids by the mushrooms shifted the degradation of the structural carbohydrates. This effect could imply on glucan concentration of the mushrooms, however glucan extent was not measured in the above mentioned study [22]. In the past, other olive mill by-products and wastes were also used as substrates for the cultivation of various edible mushrooms [23,24,25,26]. Lavi et al. [27] previously compared the types of glycoside bonds in polysaccharides harvested from *P. pulmonarius* under different growing conditions. Polysaccharides extracted from *P. pulmonarius* fruiting bodies grown on straw (FBE) were compared to the glucans extracted from mycelia produced in submerged culture (ME). We found in the abovementioned study [27] that the glucan extracted from FBE contained 85% glucose as compared to that extracted from ME contained only 64% glucose. Both FBE and ME glucans contained significant and equal amounts of galactose (8.3%). ^13^C and ^1^H NMR analyses of the FBE preparation showed mixed α-linkages and β-anomeric carbon linkages, whereas the ME polysaccharide demonstrated mainly α-glucan linkages.

The objectives of this study were: (1) to determine the glucan content, with emphasis on α glucans, in two distinct morphological sections of the fruiting body (cap and stalk) of seven different *Pleurotus* cultivars; (2) to measure the effect of relative substrate composition including OMSW on the glucan concentration of *Pleurotus* in order to delineate a novel methodology to improve the health-promoting effect of *Pleurotus*.

## 2. Results

### 2.1. The Yield of Extracted Glucans from Seven Pleurotus Strains

In studies aimed at examining the biological activity of α and β-glucans in different cell lines, it is essential to use soluble glucans in order to be supplemented into the cells’ medium. To our knowledge, this is the first study to evaluate the content of soluble α, β, and total-glucans, after discarding the insoluble fraction of the mushroom glucans. We extracted therefore, soluble glucans (as described in Methodology Section 4.3), from the most widely cultivated *Pleurotus* species: *P. ostreatus*, *P. ostreatus* var. *colombinus*, *P. pulmonarius*, *P*. *pulmonarius* var. *sajor caju*, *P. eryngii*, *P. cornucopiae*, and *P*. *salmoneostramineus*. The calculated extraction yield of soluble glucans for each mushroom strain is summarized in Table 1. The species giving maximal yield were *P. eryngii* and *P. Pulmonarius*. The total dried weight represents 10% of wet weight, i.e., 66 g dried weight comes from 660 g fresh mushrooms.

### 2.2. α-Glucan and Total Glucan Content among Seven Pleurotus Strains (Five Species)

**Figure 1:**
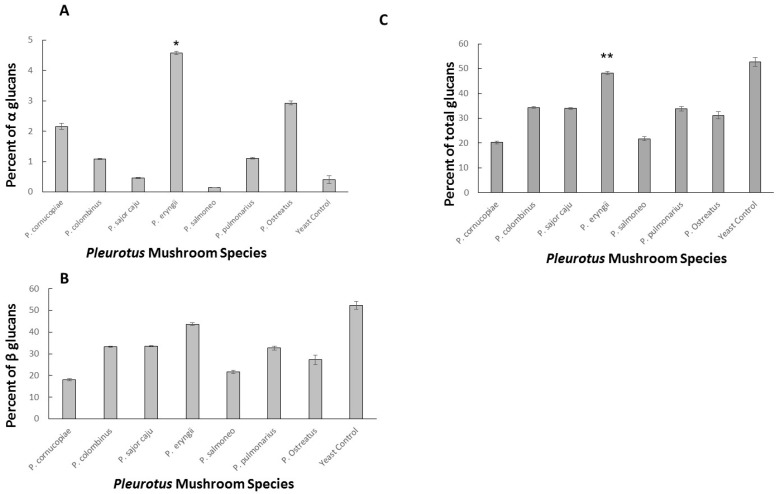
(**A**) α-glucan concentration in various *Pleurotus* strains; (**B**) β-glucan concentration in various *Pleurotus* strains and (**C**) Total glucan concentration. All concentrations are related to the percentage of dried weight. * *p* < 0.01; ** *p* < 0.05 as compared to *P. salmoneostramineus*.

Figure 1 illustrates the glucan content (α-glucan, total, and β-glucan determined by difference between total and α-glucans), determined by the Megazyme kit [28,29], in all sections of the seven dried different *Pleurotus* strains. Glucan content varied considerably among the *Pleurotus* strains. However, the maximal variability was observed in the α-glucan concentrations. As depicted in Figure 1, the total glucan content ranged from 20.25 ± 0.52% to 48.27 ± 0.68%, whereas the α-glucan content ranged from 0.47 ± 0.02% to 4.57 ± 0.06%. The mushroom species containing the higher concentration of α-glucans, β-glucans, and thus total glucans, was *P. eryngii*.

### 2.3. Total, α-Glucan, and β-Glucan Distribution in P. eryngii between Caps (C) and Stalks (S)

**Figure 2:**
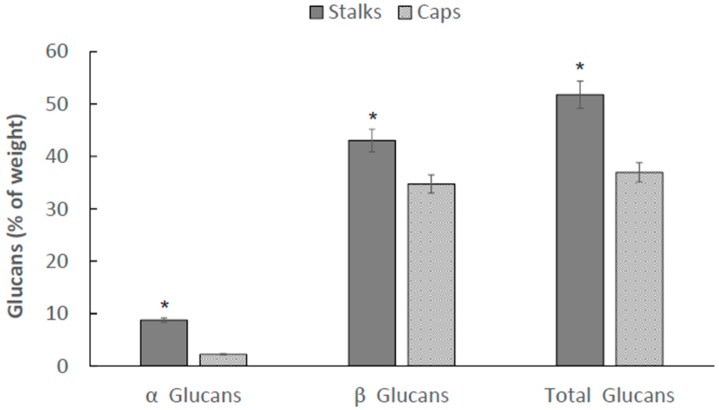
α, β, and total glucan concentrations in caps and stalks of the mushroom strain *Pleurotus eryngii*. * *p* < 0.01 stalks as compared to caps.

Since α, β, and total glucans concentrations measured in *P. eryngii* fruiting bodies were the highest, we consequently centered our attention to this specific strain. We sought to measure the distribution of glucans in *P. eryngii* between caps (C) and stalks (S). The measurements are summarized in Figure 2. The highest glucan levels (α, β, and total) are concentrated in the stalks. This finding is novel and its implications for the mushroom physiology deserves to be further investigated.

### 2.4. The Effect of Cultivation of Pleurotus Eryngii in Various Relative Amounts of Eucalyptus Sawdust and Olive Mill Solid Waste (OMSW) on Concentrations of Total Glucans, α-Glucans, and β-Glucans

We investigated several unique cultivation substrates for the growth of *P. eryngii* that use environmentally problematic agricultural waste and at the same time may enable them to synthesize higher amounts of glucans [30]. *P. eryngii* was grown in a substrate composed of a sterilized mixture of eucalyptus sawdust containing different concentrations of OMSW (from 20% up-to 80% *w*/*w*).

**Figure 3:**
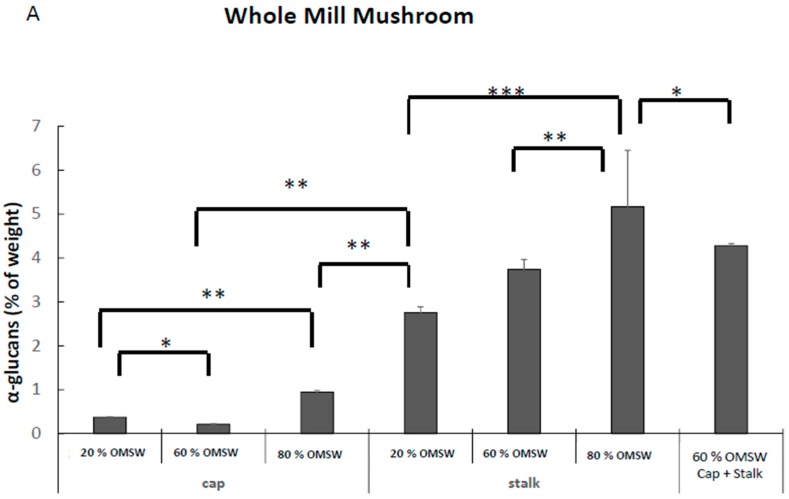

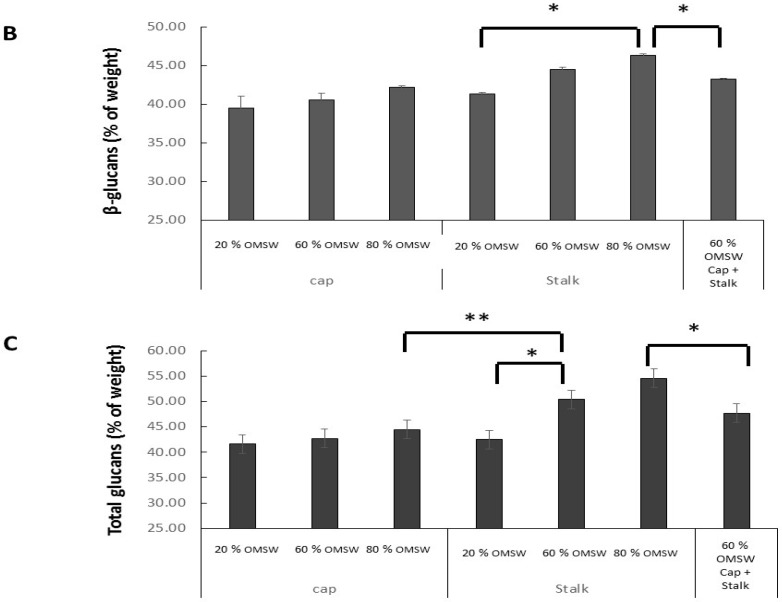
α (**A**), β (**B**), and total (**C**) glucan concentrations in whole mill mushrooms prepared from caps and stalks from *Pleurotus eryngii* mushrooms cultivated in various relative amounts of eucalyptus sawdust and OMSW. * *p* < 0.05; ** *p* < 0.01; *** *p* < 0.001.

First, we measured the effect of cultivation of *P. eryngii* in these environmental conditions on the yield of the extracted glucans fraction. There was a dose dependent effect of relative amounts of eucalyptus sawdust and OMSW on the yield of extracted glucan fraction (Table 2). Second, we measured the effect of cultivation of *P. eryngii* environment conditions on total, α-glucan, and β-glucans concentrations. Figure 3A demonstrates that the α-glucan fraction concentration was dramatically and dose-dependently enhanced in relation to OMSW concentration in the growth substrate. The effect was measurable both in caps and in stalks of the fruiting bodies, being the effects of OMSW relative amount more notorious on α-glucan concentration and preferentially the effect more accentuated in stalks than in caps.

The effect of relative concentrations of OMSW on the substrate was less pronounced for both β-glucans and total glucan (Figure 3B,C); nonetheless, 80% of OMSW still exerted the most prominent effect (Figure 3B,C).

**Figure 4:**
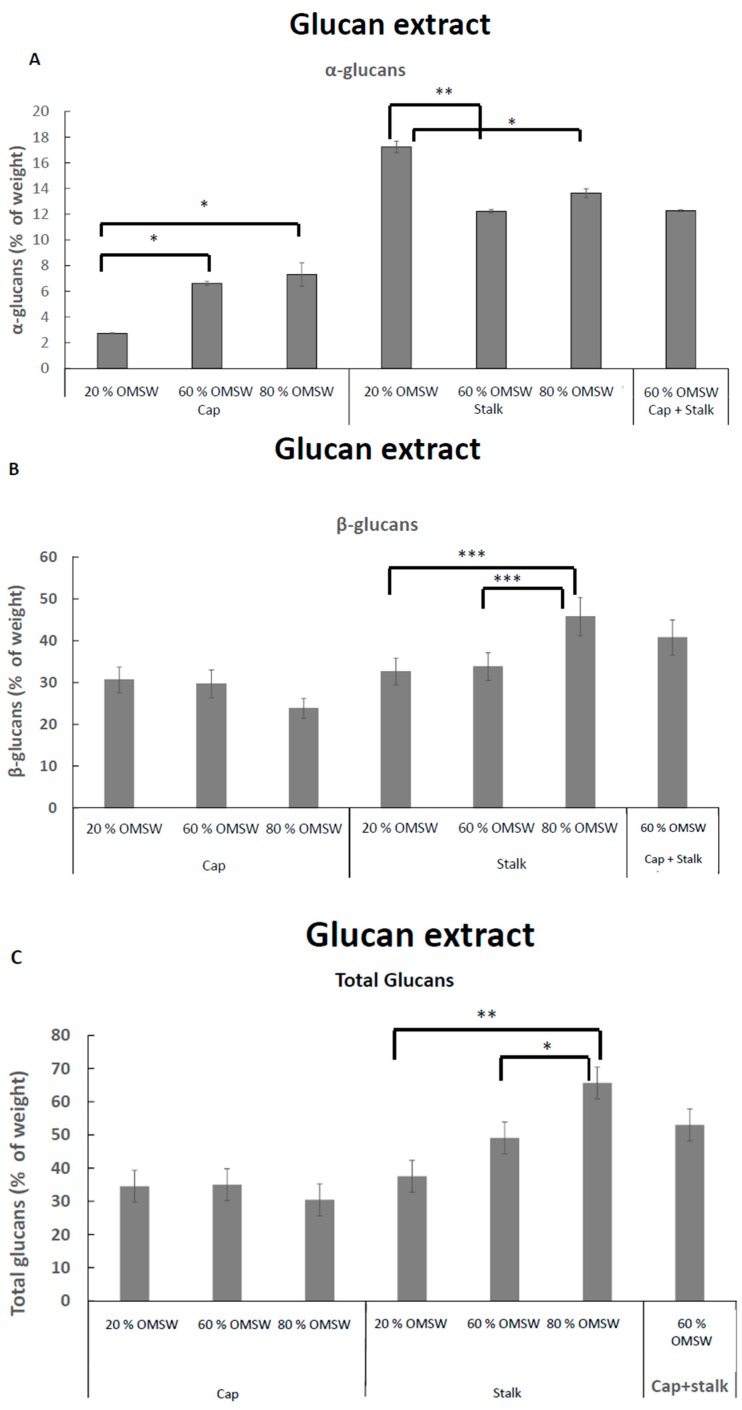
α (**A**), β (**B**), and total (**C**) glucan concentrations in glucan extracts prepared from caps and stalks from *Pleurotus eryngii* mushrooms cultivated in various relative amounts of eucalyptus sawdust and OMSW. * *p* < 0.02; ** *p* < 0.01; *** *p* < 0.05.

Third, we measured α, β, and total glucans concentrations in extracted lyophilized fractions from *P. eryngii*. The concentration of α-glucans were higher in extracted lyophilized solubilized glucans fraction from stalks (Figure 4A) as compared to caps. Overall, the net α-glucans concentrations in this extracted lyophilized fraction (see Section 4.3 of materials and methods) was higher than in mill prepared from different parts of *P. eryngii* mushrooms (Figure 3A). The concentrations in the extracted glucan fraction were generally four times higher. In stalks, the concentration in extracted glucans was eight times higher than in the whole mill prepared from *P. eryngii* stalks. Moreover, in the extracted glucan fraction, we retrieved a similar distribution of β-glucan concentrations in caps and stalks as for the whole mill. Additionally, a similar effect of OMSW concentration was obtained for β-glucan concentrations (Figure 3B vs. Figure 4B) and total glucans (Figure 3C vs. Figure 4C). The glucan concentrations are expressed as percent of extract or mill preparations.

## 3. Discussion

In this study, we present a quantitative analysis of glucan levels of five different *Pleurotus* species (seven strains). The highest α and total glucan content was observed in *P. eryngii*, with values ranging between 4.5 ± 0.40 % for α-glucans and 48.9 ± 4.82% for total glucan content. β-glucan content obtained by difference between total and α-glucans was 43.47 ± 4.21%. In contrast, *P. salmoneostramineus* exhibited the lowest glucan concentrations: 0.29 ± 0.04% for α glucans; to 17.09 ± 0.18% for β-glucan content; and 17.34 ± 0.21% for total glucan content (Figure 1). The results are consistent with previous publications [7]. However, as we tested one strain per species only, it should be mentioned that variability could exist between strains as recently shown [5]. It could consequently postulate that *P. salmoneo* would not provide a mushroom with suitable health-promoting value associated with glucan content. In contrast, we could conclude that *P. eryngii* with the highest glucan content could be used as a nutritional source of high glucan production for the modern food industry.

Once we identified the *Pleurotus* species having the highest glucan concentrations, i.e., *P. eryngii*, we measured glucans concentrations in two different sections of the fruiting body: the caps and the stalks. The separation between stalk and cap was determined as the place where the gills began to appear on the stalk. The highest glucans concentrations were consistently measured in the stalks section of *P. eryngii*. *P. eryngii* fruiting body contained relatively small amounts of α-glucans: 2.8% in caps and in contrast 9.4% in stalks. The contents of β-glucans, which were calculated as a difference between the total and α-glucans, were 39.1% in the caps and 43.7% in the stalks. It is evident that the glucan contents showed topological specificity in *P. eryngii*. The contents of these polysaccharides in the fruiting bodies vary with the strains and species. The stalks are a better source of glucans than are the gastronomically attractive cap, and, therefore, the stalks can be used for the preparation of biologically active polysaccharide complexes utilizable as food supplements, and the caps can be used for other nutrient and active components source (such as ergosterol [31]).

In the present study, we used the enzymatic methods based on the Megazyme kit© [28,29] to measure glucans concentrations. Manzi et al. [32] previously reported, about 1–2 order lower contents of β-glucan in fresh fruit bodies of *P. ostreatus* (0.24–0.38% and 1.6% fresh weight) and *P. eryngii*. (0.22–0.38% and 3.1% fresh weight) measured according to an enzymatic analysis based on direct hydrolysis by lichenase and β-glucanase. Synytsya et al. [4] suggested that the possible reasons for the lower β-glucan content measured by Manzi et al. [32,33] appears to be the occurrence of a fiber residue. This fiber residue may be, for example, an inert material such as the chitin-glucan complex, which is an insoluble material that probably prevents the diffusion of enzymes during the β-glucan determination. Support to this assumption is given by the increase of β-glucan concentration in the mushroom after cooking (disintegration of the insoluble chitin-glucan complex) or in the samples that were not washed out with the aqueous ethanol that may remove the fraction of low molecular β-glucans [34].

We consequently aimed at enhancing the glucan content in *P. eryngii*. To this end, we modulated glucans levels by cultivating the mushrooms in a substrate containing different levels of olive mill solid wastes (OMSW). Here we used the solid fraction of a three-phase olive oil mill, this substrate is different in its composition from the two-phase mill or olive pruning residues that are more toxic and were investigated in other studies [23]. The reason we selected this strategy is based on the previous study by Reverberi et al. [30], who found that growing *Pleurotus* on substrate containing OMSW greatly stimulated the β-1,3-glucan synthase activity of *P. ostreatus*. Reverberi et al. [30] claimed that a mechanism based on response to oxidative stress could act as a switch for the β-glucan biosynthesis in *Pleurotus*. In fact, oxidative stress may occur in the presence of OMSW, due to either the presence of oxidizing phenol compounds or the reactive oxygen species formed during laccase activity [30]. In fact, many fungal β-glucans show an antioxidant activity, as reported for gluconoxylomannan in *Tremella* spp. and lentinan in *L. edodes* [30]. OMSW could also act as a source of nitrogen, minerals, and other nutrients favoring a strong development of the mushrooms as sawdust is a poor medium. Fungal cultivation on OMSW, which is considered as hazardous waste, could have a double effect: the degradation and utilization of agricultural wastes while producing pharmacologically active compounds. In this regard, Dawoud [34] recently demonstrated that inducing physical stress factors (such as γ irradiation) profoundly affects the fungal growth and synthesis of the biologically and pharmaceutically active polysaccharide 1,3-β-glucan, and concomitantly affects the 1,3-β-glucan synthase enzyme activity in submerged culture of *P. ostreatus*. Additionally, Elisashvil et al. [35], demonstrated that carbon and nitrogen source affects mycelial basidiomycetes exopolysaccharide production in submerged culture. It seems that fungal cell walls can be reinforced by nutritional and environmental signals or stress. This can be demonstrated by increased resistance to glucanases and other cell-wall-degrading agents, but also to heat stress [36,37].

In our study, the *P. eryngii* grown on sterilized mixture of eucalyptus sawdust and OMSW at different concentrations responded in the upregulation of the levels of all glucans, being minimal at 20%, median at 60%, and maximal at 80%. The effects were more distinct in stalks than in caps. The effect of α-glucans has been associated with increased insulin sensitivity and improved insulin resistance in peripheral target tissues, therefore they have been demonstrated to induce an anti-diabetic effect [38]. These mushrooms could be used therefore, as an excellent nutritional source containing high glucan concentrations with health-promoting activity to be used by the modern food industry as excellent functional foods.

## 4. Materials and Methods

### 4.1. Mushroom Growth Conditions

The *Pleurotus* mushroom species and strains used in this study are summarized in Table 3. The mushrooms were grown using a standard system under standardized, controlled conditions typical of commercial operations and would therefore be representative of mushrooms available to consumers.

The mushrooms were grown on a sterilized mixture of eucalyptus sawdust and olive mill solid waste (OMSW). The OMSW is the solid fraction from a three-phase olive mill. The olive mill waste-water was discharged. OMSW was added at concentrations from 20% (*w*/*w*) up to 80% (*w*/*w*). The mixture was wetted to 51% water content and packed into 15 L polypropylene bags, containing a microporous filter, 2 kg wet substrate/bag. The bags were autoclaved at 121 °C for 1 h, closed and cooled to 25 °C for inoculation with spawn. The culture was incubated at 25 °C for 14–21 days. For fruiting, the bags were opened and the temperature was reduced to 16 °C with a relative humidity of 90%, 12 h daily light and CO_2_ concentration of 600–800 ppm.

### 4.2. Mushroom Sample Collection and Preparation

Fruiting bodies were freshly harvested at the Matityahu Experimental Farm (Upper Galilei, Israel). Fruiting bodies were collected according to their cap opening and their cap color. After harvest, fruiting bodies were weighed; dried and whole fruiting bodies, stalks, or caps samples were milled to pass a 1.0 mm screen using a Retsch centrifugal mill preparation unit in order to allow for glucan analyses.

The proportion of stalks and caps in wet and dried *Pleurotus eryngii* mushrooms cultivated in 80% and 20% OMSW are summarized in Supplemental Table S1. The percent of dried weight preparations from stalks and caps grown in 20%, 60%, and 80% OMSW are shown in Supplemental Table S2. Stalks yielded more dried matter than caps.

### 4.3. Preparation and Extraction of Glucans from Different Powdered Dried Mushroom Strains

Dried powder from the different mushroom strains was prepared as mentioned in the previous section. Each 100 g of powdered fruiting bodies originated from each of the strains was extracted with 1000 mL dH_2_O at 121 °C for 30 min (in autoclave). The extract was then centrifuged at 13,000× *g* at 10 °C for 15 min. Ethanol (EtOH) was added to the supernatant, to a final concentration of 67% (*v*/*v*), and the mixture was stored overnight at 20 °C. The float was taken out, and the viscous fraction was removed and lyophilized and glucan content analyzed on the lyophilized material.

### 4.4. Glucans Analysis

The glucan content of the dried *Pleurotus* fruiting bodies (Section 4.2) or extracted glucans (Section 4.3) was determined using a mushroom and yeast specific β-glucan kit (Megazyme International, Wicklow, Ireland) based on a colorimetric reaction [28,29]. The analysis was conducted according to the manufacturer’s instructions. The enzyme kit contains exo-1,3-β-glucanase, β-glucosidase, amyloglucosidase and invertase; glucose determination reagent (glucose oxidase peroxidase, and 4-aminoantipyrine), and glucose standard solution. Total-glucan, α-glucan, and β-glucan were measured in the above-mentioned samples.

Measurement of total glucan content is performed following a previous solubilization in ice cold 12 M H_2_SO_4_ of the *Pleurotus* dried samples and then hydrolysis to near completion in 2 M H_2_SO_4_ for 2 h at 100 °C. Subsequent to neutralization with 2 M potassium hydroxide, glucose hydrolysis is accomplished using a mixture of exo-1,3-β-glucanase and β-glucosidase in sodium acetate buffer (pH 5.0) for 1 h at 40 °C. The absorbance of the resulting color complex was measured at 510 nm using a spectrophotometer (Synergy 2, Multi-Mode Reader, BioTek, Winooski, VT, USA). Total Glucan (% *w*/*w*) and α-Glucan (% *w*/*w*) were calculated, and by difference between those two, the β-glucan content (% *w*/*w*) was finally calculated. Glucan content was expressed as percentage (*w*/*w*) of the fruiting body dry weight. The control provided by the kit consists of glucans extracted from the yeast *Saccharomyces cerevisiae* [28,29].

### 4.5. Statistics

Analyses of the experimental data was performed using the two-way ANOVA test. All data are expressed as mean ± standard deviation (SD). The level of statistical significance was set at *p* < 0.05.

## 5. Conclusions

The present study demonstrate that the edible mushrooms *Pleurotus eryngii* can provide a rich source for glucan extraction (specially concentrated on stalks) for nutritional and medicinal applications and that glucan content in mushroom fruiting bodies can be further enriched by applying OMSW into the cultivation substrate.

## Figures and Tables

**Table 1:** Calculated yield extraction of total glucans/dry matter, for different mushroom strains.

Mushroom Species	Total Dried Weight (g)	Glucans Extracted (g)	Yield %
*Pleurotus ostreatus*	66	2.64	4
*Pleurotus ostreatus* var. *colombinus*	58	1.74	3
*Pleurotus pulmonarius*	70	2.80	4
*Pleurotus sajor caju*	74	1.48	2
*Pleurotus eryngii*	55	3.30	6
*Pleurotus cornucopiae*	64	1.92	3
*Pleurotus salmoneostramineus*	86	2.58	3

The mushroom giving the maximum yield of glucans extracted were *Pleurotus eryngii* (6%).

**Table 2:** The effect of cultivation of *Pleurotus eryngii* mushrooms in various relative amounts of eucalyptus sawdust and olive mill solid waste (OMSW) on yield of preparation of extracted glucans fraction.

Treatment (% OMSW)	Yield (%)
Control (100% eucalyptus sawdust)	6.2 ± 0.39
20	12.56 ± 1.39
60	24.6 ± 4.23
80	29.24 ± 6.65

There was a dose dependent effect of relative amounts of eucalyptus sawdust and OMSW on the yield of extracted glucan fraction from dried mushrooms stalks.

**Table 3:** The names of the species and strains of the different mushrooms used in the present study.

Mushroom Name	Commercial Strain
*Pleurotus ostreatus*	Fungisem K-12
*Pleurotus ostreatus* var. *colombinus*	Sylvan 3030
*Pleurotus pulmonarius*	Sylvan 3014
*Pleurotus pulmonarius* var. *sajor caju*	Jamaica 503
*Pleurotus eryngii*	Mycelia 2600
*Pleurotus cornucopiae*	Sylvan 3040
*Pleurotus salmoneostramineus*	Mycelia 2708

## Supplementary Materials

Supplementary materials can be found at www.mdpi.com/1422-0067/18/7/1564/s1.
