# Supplementary material for: Olive Mill Waste Enhances α-Glucan Content in the Edible Mushroom Pleurotus eryngii
Source: Int J Mol Sci. 2017 Jul 18;18(7):1564. doi: 10.3390/ijms18071564 (PMC5536052; doi:10.3390/ijms18071564)
Supplement: Supplementary file 1 [file ijms-18-01564-s001.pdf]

Table S1. Weight ratio between caps and stalks *in Pleurotus Eryngii.*

Treatment	Caps (Wet Weight)	Stalks (Wet Weight)	Caps (Dried Weight)	Stalks (Dried Weight)
Pleurotus Eryngii (80% OMSW)	80.9	19.1	67	33
Pleurotus Eryngii (20% OMSW)	81.1	18.9	69	31

Table S2. Percent of dried weight obtained from *Pleurotus Eryngii* caps and stalks.

Treatment	Percent of dried Weight (Caps)	Percent of Dried Weight (Stalks)
Pleurotus Eryngii (80% OMSW)	10.5	17.9
Pleurotus Eryngii (60% OMSW)	8.9	16.1
Pleurotus Eryngii (20% OMSW)	9.8	16.3
